# Dual-ratio approach for detection of point fluorophores in biological tissue

**DOI:** 10.1117/1.JBO.28.7.077001

**Published:** 2023-07-22

**Authors:** Giles Blaney, Fernando Ivich, Angelo Sassaroli, Mark Niedre, Sergio Fantini

**Affiliations:** a Tufts University, Department of Biomedical Engineering, Medford, Massachusetts, United States; b Northeastern University, Department of Bioengineering, Boston, Massachusetts, United States

**Keywords:** Monte-Carlo methods, fluorescence, autofluorescence, signal-to-noise ratio, flow-cytometry, dual ratio/dual slope

## Abstract

**Significance:**

Diffuse *in vivo* flow cytometry (DiFC) is an emerging fluorescence sensing method to non-invasively detect labeled circulating cells *in vivo*. However, due to signal-to-noise ratio (SNR) constraints largely attributed to background tissue autofluorescence (AF), DiFC’s measurement depth is limited.

**Aim:**

The dual ratio (DR)/dual slope is an optical measurement method that aims to suppress noise and enhance SNR to deep tissue regions. We aim to investigate the combination of DR and near-infrared (NIR) DiFC to improve circulating cells’ maximum detectable depth and SNR.

**Approach:**

Phantom experiments were used to estimate the key parameters in a diffuse fluorescence excitation and emission model. This model and parameters were implemented in Monte Carlo to simulate DR DiFC while varying noise and AF parameters to identify the advantages and limitations of the proposed technique.

**Results:**

Two key factors must be true to give DR DiFC an advantage over traditional DiFC: first, the fraction of noise that DR methods cannot cancel cannot be above the order of 10% for acceptable SNR. Second, DR DiFC has an advantage, in terms of SNR, if the distribution of tissue AF contributors is surface-weighted.

**Conclusions:**

DR cancelable noise may be designed (e.g., through the use of source multiplexing), and indications point to the AF contributors’ distribution being truly surface-weighted *in vivo*. Successful and worthwhile implementation of DR DiFC depends on these considerations, but results point to DR DiFC having possible advantages over traditional DiFC.

## 1 Introduction

Diffuse *in vivo* flow cytometry (DiFC) is an emerging optical technique that enables fluorescence detection of rare circulating cells in the bloodstream in the optically diffusive medium.^1^^–^^4^ Dual slope or dual ratio (DR) is a new diffuse optical technique that is designed to suppress noise in the optical signal and reduce sensitivity to superficial tissue regions.^5^^–^^8^ A challenge of DiFC is the contamination of the target fluorescence signal from noise, which may be associated with background autofluorescence (AF). In this work, we investigate the possibility of utilizing DR techniques to suppress this noise and AF, thus enabling better signal-to-noise ratio (SNR) of DiFC measurements.

### 1.1 Diffuse In Vivo Flow Cytometry

In DiFC, the tissue surface is illuminated with laser light, typically delivered by an optical fiber bundle. As fluorescently labeled circulating cells pass through the DiFC field of view, a transient fluorescent peak may be detected at the surface using a collection fiber. Since DiFC uses diffuse light, it is possible to detect circulating cells from relatively deep-seated and large blood vessels several mm into tissue. Hence, the key advantage of DiFC is that it allows for the interrogation of relatively large circulating blood volumes enabling the detection of rare cells.

For example, a major application of DiFC has been in mouse cancer research. Circulating tumor cells (CTCs) migrate from solid tumors, move through the circulatory system, and may form secondary tumor sites. As such, CTCs are instrumental in hematogenous cancer metastasis and are a major focus of medical research. However, CTCs are extremely rare and frequently number fewer than 100  CTC/mL of blood in metastatic patients.^9^ In the clinic, the current gold standard method to study CTCs is via liquid biopsy (blood draws) using the Food and Drug Administration cleared system CellSearch.^10^^,^^11^ For example, in breast cancer patients, ≥5  CTCs detected in 7.5 mL blood samples with CellSearch is associated with poor cancer prognosis.^9^ However, the small amount of blood analyzed (0.015% of the total peripheral blood volume) is known to yield poor sampling statistics, even assuming ideal Poisson statistics.^12^ Moreover, short-term temporal fluctuations of these cells may cause errors in estimating the CTC numbers based on when blood was drawn.^13^ Thus DiFC to enumerate CTCs continuously and non-invasively in large circulating blood volumes *in vivo* may help address these limitations. In mouse xenograft tumor models, we have previously performed DiFC on the mouse tail above the ventral caudal artery. These studies showed that it is possible to non-invasively sample the entire peripheral blood volume in ∼15  min, permitting the detection of very rare CTCs.^2^^,^^14^

**Fig. 1:**
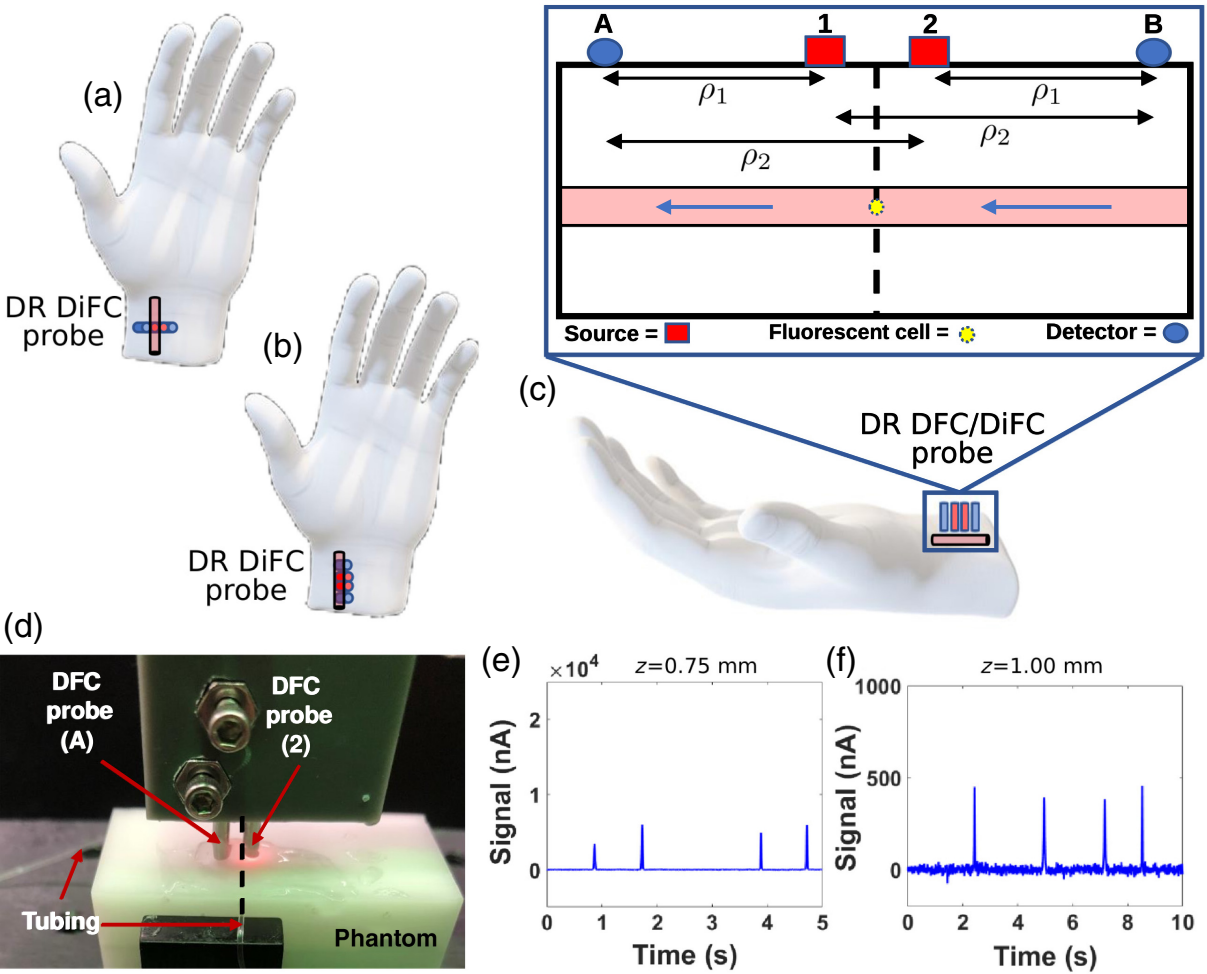
Conceptual application of DR DiFC. In principle, source and detector pairs could be arranged (a) perpendicular or (b) parallel to the underlying artery (in this case, the radial artery). (c) Use of two sources (1 and 2) and two detectors (A and B) would permit four source and detector pairs separated by a source–detector distance (ρ). (d) Photograph of NIR DiFC system^3^ on a diffusive flow phantom with fiber probes arranged perpendicular to the tubing direction. The dashed black line in (d) corresponds to the mid-plane shown in (c). The instrument permitted measurement with a single source and detector pair, in this case (2 and A). Background-subtracted sample DiFC data of fluorescent microspheres embedded (e) 0.75 mm deep and (f) 1.00 mm deep in a phantom with a flow channel.

Moreover, if paired with specific molecular contrast agents for CTCs (e.g., as those for fluorescence-guided surgery), translation of DiFC to humans may be possible.^15^^–^^17^ We recently demonstrated that CTCs could be labeled directly in the blood of mice using a folate-targeted fluorescent target (EC-17) and be detected externally with DiFC.^18^ However, two major technical challenges exist when applying DiFC to humans. First, the measured DiFC signal combines a fluorescence signal from the circulating cell and a non-specific background AF signal from the surrounding tissue. Although the non-specific background signal can be subtracted, noise contributed from the background cannot be removed. As such, the noise may obscure small-amplitude fluorescence signals. To illustrate this SNR consideration, example DiFC data showing signal peaks from detected flowing fluorescent microspheres embedded 0.75 and 1.00 mm deep in a phantom are shown in Figs. 1(e) and 1(f), respectively. Note that each peak represents individual mirco-spheres passing through DiFC field-of-view. The second technical challenge relates to the depth of blood vessels in humans. Suitable blood vessels, such as the radial artery in the wrist [Figs. 1(a)–1(c)], are expected to be about 2 to 4 mm deep (i.e., significantly deeper than in mice).^19^ Alignment to the radial artery can be potentially achieved in humans by placing DiFC probes in the “volar” wrist beneath the thumb, between the wrist bone and the tendon. Some people may be able to observe the vessel through the skin, facilitating alignment. Small misalignments (∼1  mm) do not significantly affect the signal quality because of the wide sensitivity volume of diffuse light. We recently showed that individual cell fluorescence signals might be detectable up to 4 mm deep using near-infrared (NIR) fluorophores and a source–detector distance (ρ) of ∼3  mm, increased depth and larger relative non-specific background signals presents challenges for detection of weakly labeled CTCs.^20^ Hence methodology for reduction of the background signal and its contributed noise, such as dual slope or DR described in Sec. 1.2, would be extremely valuable for potential human translation of DiFC.

### 1.2 Dual Ratio

One of the most basic measurements in diffuse optics is that of the single-distance (SD) reflectance (R), which is measured by placing a point source and a point detector on the surface of a highly scattering medium. In practice, the detector does not measure the theoretical R but instead, some optical intensity (I) that is proportional to the theoretical R (Sec. 7.2.1). The proportionality of I to R is controlled by coupling coefficients (C’s), which may be associated with the coupling efficiency of the optodes (sources or detectors) with the medium as well as any other multiplicative factor on R.

Recently, we introduced new measurement types that attempt to represent the theoretical R by canceling any C’s associated with optodes. These are the dual slope^7^ or the DR^8^ data types, which in their calculation cancel any multiplicative factors associated with optodes.^8^^,^^21^ These measurement types essentially recover the spatial dependence of R over multiple ρ’s. This is achieved from a symmetrical optode arrangement of sources and detectors, such as the one in Fig. 1(c).

A further advantage of the dual-slope or DR data type is the reduced contribution to the signal from superficial parts of the diffuse medium.^5^^–^^7^ With the hypothesis that the unwanted AF contributors in DiFC measurements are mainly near the surface, we decided to explore if these data types could suppress the AF component of the DiFC measurement. We hypothesize that this AF suppression, together with the cancellation of noise through the cancellation of C’s, will improve the SNR of DR over traditional SD DiFC measurements using NIR optical wavelengths (λ’s). This improved SNR of DR DiFC could enable the detection of deep fluorescent targets, which is a current struggle of DiFC as discussed in Sec. 1.1. Therefore, this work investigates the potential of DR in the DiFC application and identifies the key parameters that control whether or not DR will be advantageous.

## 2 Methods

### 2.1 Proposed Dual-Ratio Signal

Most diffuse optical measurements recover an optical signal using a source and a detector, often each placed on a tissue surface. We consider each measurement of I with a source and detector as a SD measurement (Sec 6.1). Previous DiFC work has considered these types of measurements;^20^ however, in this work, we introduce a DiFC measurement type based on a combination of SDs to form a DR (Sec. 6.3).^7^^,^^8^

DR is defined in detail in Sec. 6.3 and will be summarized here. The DR measurement is defined as follows:

$$\mathrm{DR}=\sqrt{\frac{I_{l,I}I_{l,\mathrm{II}}}{I_{s,I}I_{s,\mathrm{II}}}},$$ (1)

where the l and s subscripts represent I measurements at long or short ρ’s, respectively. Additionally, the I and II subscripts show whether it is the first or second symmetric I measurement in a DR. The exact geometric requirements for I and II are described in more detail in Sec. 6.3 and previous publications (where DR is referred to as dual slope, since geometric requirements for the two are the same).^22^ For this work, consider Fig. 1(c). In this case, s, I and l, I are detector A and source 1 (SD A1) and SD A2, respectively. And similarly, for detector B, s, II and l, II are SD B2 and SD B1, respectively.

### 2.2 Monte-Carlo Models

This work presents simulation results based on a Monte-Carlo (MC) model run in Monte-Carlo Extreme (MCXLAB 2020 running in MATLAB 2023a on an NVIDIA RTX 4090).^23^ For all simulations, optical properties meant to represent λ of 810 nm were used.^20^ These were an absorption coefficient $\left(\mu_{a}\right)$ of $0.002\;{\mathrm{mm}}^{-1}$, a scattering coefficient $\left(\mu_{s}\right)$ of $7\;{\mathrm{mm}}^{-1}$, an anisotropy factor (g) of 0.9, and an index of refraction (n) of 1.37. The MC was run by launching $1\times 10^{9}$ photons into a 30  mm×30  mm×30  mm volume, with grid spacing of 0.1 mm and 10 time gates ending at 10 ns. To calculate the continuous wave R, the MC’s time-domain R was integrated over the time gates.

For the coordinate system, the surface of the medium was considered to be z=0  mm (positive z pointing into the medium), and the center (with the edges 15 mm away in each direction) to be x=0  mm and y=0  mm. Using this coordinate system, detector A was placed at −3.5 x^  mm, detector B at 3.5 x^  mm, source 1 at −0.5 x^  mm, and source 2 at 0.5 x^  mm. In the case of the 0 mm ρ simulation, both source and detector were placed at the origin. A separate MC was run for each of these optodes to find the fluence rate (Φ) distribution for a MC source placed at the optode position. This follows the adjoint method to calculate the R for a given SD set.^24^ In the case of sources, a MC pencil beam was used, and in the case of detectors, a MC cone beam with a numerical aperture of 0.5 was used to simulate the collection geometry of a fiber more accurately. This type of source distribution associated with the detectors is done to be closer to the condition of validity of the reciprocity theorem, which is crucial for applying the adjoint method. These Φ’s were used with Eq. (15) to yield the fluorescence Jacobian (W),^20^ a key parameter used in all the simulation results as described in Appendix B.

### 2.3 Experimental Measurements with Diffuse Flow Cytometry

#### 2.3.1 Phantom experiments with fluorescent microspheres in-vitro

To estimate the potential performance of a DR DiFC instrument, we performed DiFC measurements in tissue-mimicking flow phantoms with NIR wavelengths (770 nm excitation and 810 nm emission).^20^ Briefly, we used Jade Green high intensity (Spherotech Inc., Lake Forest, Illinois, United States) cell-mimicking fluorescent microspheres (size ∼10 to 14  μm) for NIR SD measurements.^3^ Microspheres were suspended at a concentration of $1\times 10^{3}\;{\mathrm{mL}}^{-1}$ in phosphate buffered saline and flowed through Tygon tubing (internal diameter of 0.25 mm; TGY-010-C, Small Parts Inc., Seattle, Washington, United States) embedded in a tissue-mimicking optical flow phantom made of high-density polyethylene. Microsphere suspensions were pumped with a syringe pump (0-2209, Harvard Apparatus, Holliston, Massachusetts, United States) at a flow rate of $50\;\mu L\;\min^{-1}$ or average flow velocity of $17\;\mathrm{mm}\;s^{-1}$ to approximate linear velocities in the mouse leg femoral artery.^2^^,^^25^ The tubing at a depth of 1.50 mm in the phantom.

We performed SD DiFC measurements using a ρ of 3 mm [Fig. 1(c) $\rho_{1}$]. Two fiber bundle probes were used, one as a source fiber and the other as a detector fiber. We collected DiFC data for the source (labeled as numbers) and detector (labeled as letters) pairs A1 and B2. To test the effect of the geometric arrangement of the probes relative to the flow tube, we performed measurements in the perpendicular or parallel directions as in Figs. 1(a) and 1(b). For illustration, a photograph of the A2 (at $\rho_{2}=4\;\mathrm{mm}$) SD NIR DiFC measurement is included in Fig. 1(d), which shows a perpendicular placement of the probes to the tubing.

We collected 15 min DiFC scans and processed the data as described previously.^1^ First, we performed background subtraction using a 5 s moving median window. Transit fluorescent peaks were measured as fluorescent microspheres were detected with the DiFC system. Here peaks were defined as having a minimum amplitude of five times the noise (standard deviation of the data in a 1 min moving window). We analyzed peak amplitude, width (i.e., shape), and noise properties. These parameters were used in Appendix C to find the important parameters of fluorescence efficiency (η) and fluorescent $\mu_{a}$ needed to simulate SNR of DiFC measurements from the MC output.

#### 2.3.2 Measurement of in vivo autofluorescence on mice

As discussed in more detail in Sec. 4.1 and Sec. 8.1.1, knowledge of the distribution of sources of AF is necessary for building a computational model of DR DiFC noise and background. To address this, we measured the AF background DiFC signal of the shaved inner thigh of recently euthanized mice: (1) at the surface of the skin and (2) at the surface of the exposed underlying muscle (without skin).

We used N=4 female BALB/c mice on a low-fluorescence chow diet for this experiment. The hair of the inner left thigh of these euthanized mice was removed with depilatory cream (Nair, Church & Dwight Co., Inc., Ewing, New Jersey, United States). The skin of the right inner thigh was surgically removed, exposing the underlying muscle. We used our red DiFC instrument^1^ to measure the background tissue AF background SD signal in both cases. During these measurements, ultrasound gel was applied between the DiFC probe and the tissue surface to minimize the index of refraction mismatch. DiFC was performed for 15 min, and the mean background (i.e., with and without skin) was calculated from the resulting data.

## 3 Results

### 3.1 Monte-Carlo Simulated Maps and Signals

**Fig. 2:**
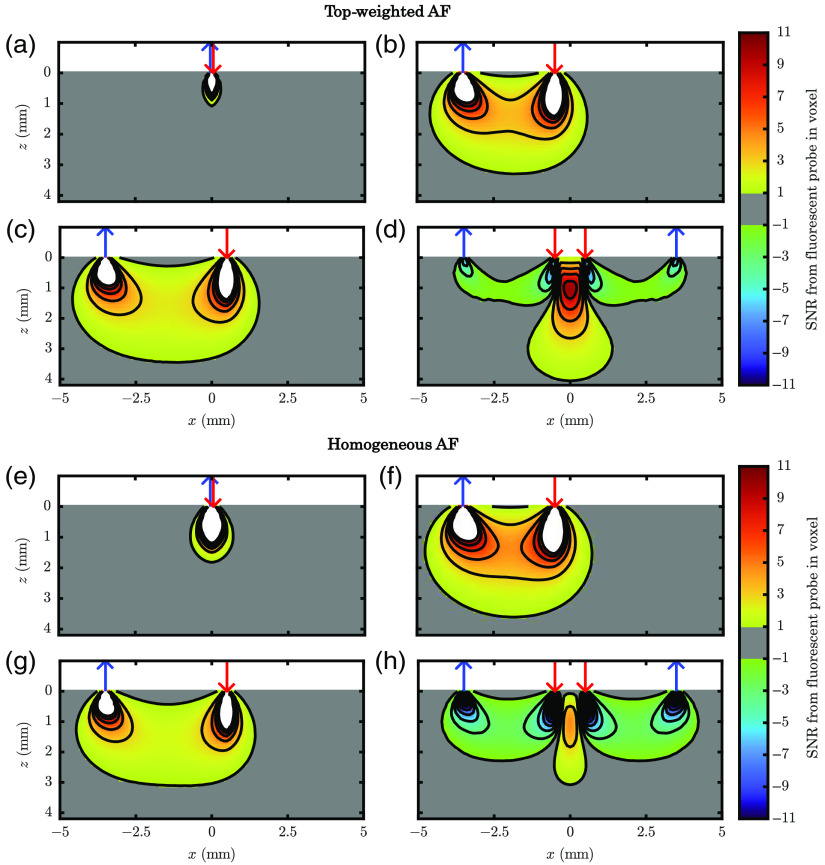
Map of the SNR to a fluorescent target at a particular position (in the y=0  mm plane) within a medium with (a)–(d) surface-weighted or (e)–(h) homogeneous AF contributors for four different measurement types with detectors represented by blue arrows and sources by red arrows. (a), (e) SD at a source–detector distance (ρ) of 0 mm. (b), (f) SD at a ρ of 3 mm. (c), (g) SD at a ρ of 4 mm. (d), (h) DR containing ρ of 3 and 4 mm. Note: white regions represent SNR greater than the maximum color-bar scale (i.e., 11), and gray regions represent absolute SNR<1. Parameters: source = pencil, detector = 0.5 NA cone, voxel=0.1  mm×0.1  mm×0.1  mm, absorption coefficient $(\mu_{a})=0.002\;{\mathrm{mm}}^{-1}$, scattering coefficient $(\mu_{s})=7\;{\mathrm{mm}}^{-1}$, anisotropy factor (g)=0.9, index of refraction (n)=1.37, for (a)–(d) surface-weighted AF fluorescence efficiency $(\eta )\propto e^{\ln(0.5)z/0.1\;\mathrm{mm}}$ for (e)–(h) homogeneous η constant, and signal and noise parameters found in Appendix C. (In this simulation, we assumed 5% NC noise.)

In Fig. 2, we show the simulated SNR from a fluorescent target placed anywhere on the y=0  mm plane. This shows that possible target positions would yield a measurable signal (SNR>1) and how strong the signal would be. One striking result that may be drawn from comparing the overall extent of the region with SNR>1 for the case with surface-weighted contributions of AF [Figs. 2(a)–2(d)] and the case with homogeneous contributions [Fig. 2(e)–2(h)]. In general, the case of homogeneous contributions of AF favors the shorter ρs. It also severely weakens the ability of DR to measure targets when they are deep within the medium. In fact, close examination of Figs. 2(f) and 2(g) suggests that, counter-intuitively, a shorter ρ can measure deeper. However, this is only evident in the homogeneous AF contributors case and not in the surface-weighted case. Thus, telling us that the advantages of one measurement type over another depend on the spatial distribution of AF contributors.

We may also compare the different ρs and the SD versus DR. For a 0 mm ρ, we see a small bulb shape close to the surface, confirming what is expected for a co-localized source and detector. The non-zero ρs for SD show the typical banana shape expected in diffuse optics but with some differences. These differences arise from the small length scale used in these simulations, on the order of 1 mm so that the light does not act fully diffuse. For this reason, the MC source model matters much for the shape of the banana. To be more realistic, we modeled the source as a pencil beam and the detector as a cone with 0.5 numerical aperture. This resulted in one side of the banana (the one near the source) being deeper than the other since the pencil beam could more effectively launch photons along the z direction into the medium. These observations show that the MC source condition simulated matters and should match reality.

Focusing on DR, we see that it can measure deeper than every other measurement in the surface-weighted AF contributors case. However, in the homogeneous case, it does no better than the long (ρ of 4 mm) SD. This is primarily because DR focuses on canceling out signals from close to the surface. Therefore, it is advantageous when the confounding signal’s contributors, the AF’s chromophore concentration or efficiency, is surface weighted. This further reinforces the importance of understanding the distribution of AF in a realistic measurement case, like tissue, since it heavily affects which measurement type is preferable when detecting deep fluorescent targets.

**Fig. 3:**
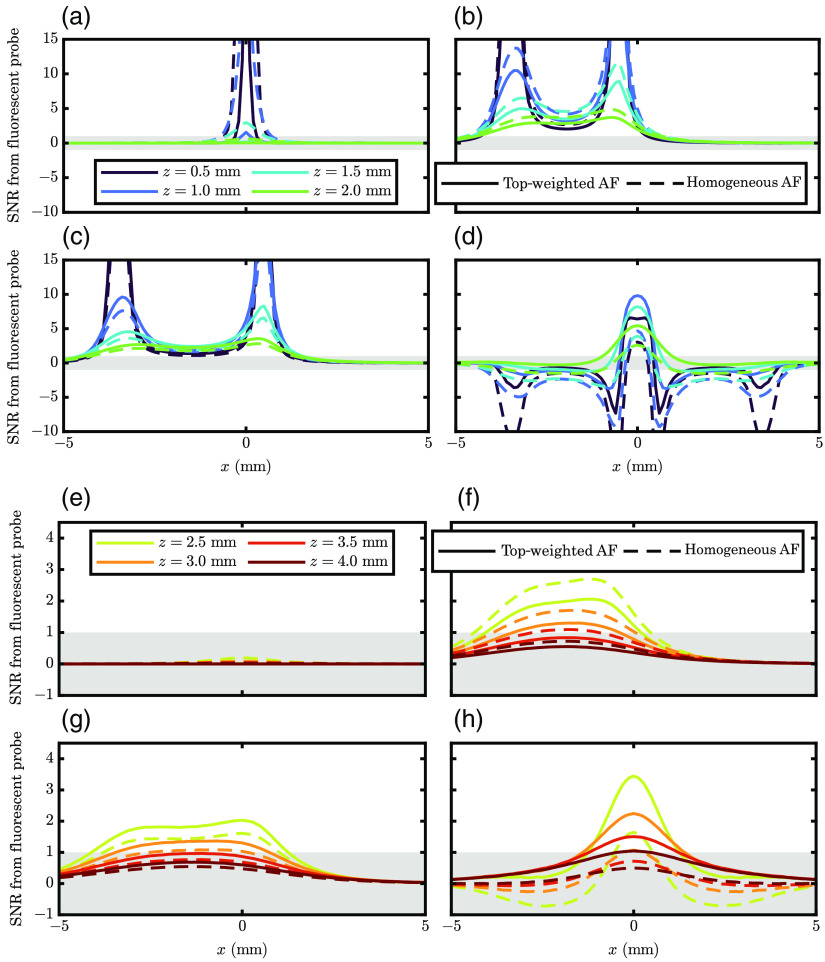
Traces of expected SNR from a fluorescent target flowing at a particular depth (color) beneath the source–detector arrangement (same as Figs. 1 and 2, y=0  mm). These results are shown for surface-weighted and homogeneous AF contributors (line-type). (a), (e) SD at a source–detector distance (ρ) of 0 mm; (b), (f) SD at a ρ of 3 mm. (c), (g) SD at a ρ of 4 mm; and (d), (h) DR containing ρ’s of 3 and 4 mm. Note: gray regions represent absolute SNR<1. Parameters: the same as Fig. 2 with assumed 5% NC noise.

In Fig. 3, we present the expected signal profiles (in SNR) that one would measure if a fluorescent target flowed parallel to the source–detector line (in the y=0  mm plane). The x axis shows the x coordinate; in an actual DiFC measurement, this would be time with the x position scaled by the velocity. Different colors represent targets flowing at different depths, and line type shows the type of distribution of contributors to AF. The gray region shows where the absolute SNR is <1, so if the curve drops into this region, we may say it is not measurable.

Many of the conclusions drawn from Fig. 3 are similar to the ones that one may draw from Fig. 2 since these traces (Fig. 3) are simply line scans of Fig. 2. So we focus on more apparent features in these traces than the map. The first is the shape of a non-zero ρ SD measurement. This is that of a double peak, which arises when the target first flows under the detector, making a strong signal, then under the source causing the target to make another strong signal. This is especially evident with shallow depths but is slightly present even in the broad traces simulated from deep zs. Further, because of the different MC source conditions, the peak height when the target passes under the source is higher than when the target passes under the detector. Therefore, we may say that this model predicts that a non-zero ρ SD measurement will produce a rather unique signal, with a double peak and a more significant peak height when the target passes under the source.

Finally, lets examine the shape of the simulated DR signal [Figs. 3(d) and 3(h)]. In this case, the signal has positive and negative components. Here it is essential to understand that these traces are differences from a baseline measurement, so that Figs. 3(d) and 3(h) show the baseline DR subtracted from the current DR. This is detailed in Appendix A and Eq. (8). Therefore negative values in the DR signal mean that the current DR value is less than the baseline value. Since one wishes to identify the presence of a fluorescent target, it does not matter whether the signal is positive or negative, so even SNR of DR<−1 may be considered a signal which can identify the target. Knowing this, we see that the DR signal is unique and could further help identify if a target is genuinely detected. The DR signal is expected first to decrease as the target flows under the detector, then an increase when the target is under the source, followed by a decrease when the target finally flows under the second detector. Therefore, these results suggest that the DR (as well as the SD) signal has a unique shape that may be used to identify a true positive detection of the fluorescent target.

### 3.2 Comparison of Phantom Experiments and Simulated Signals

**Fig. 4:**
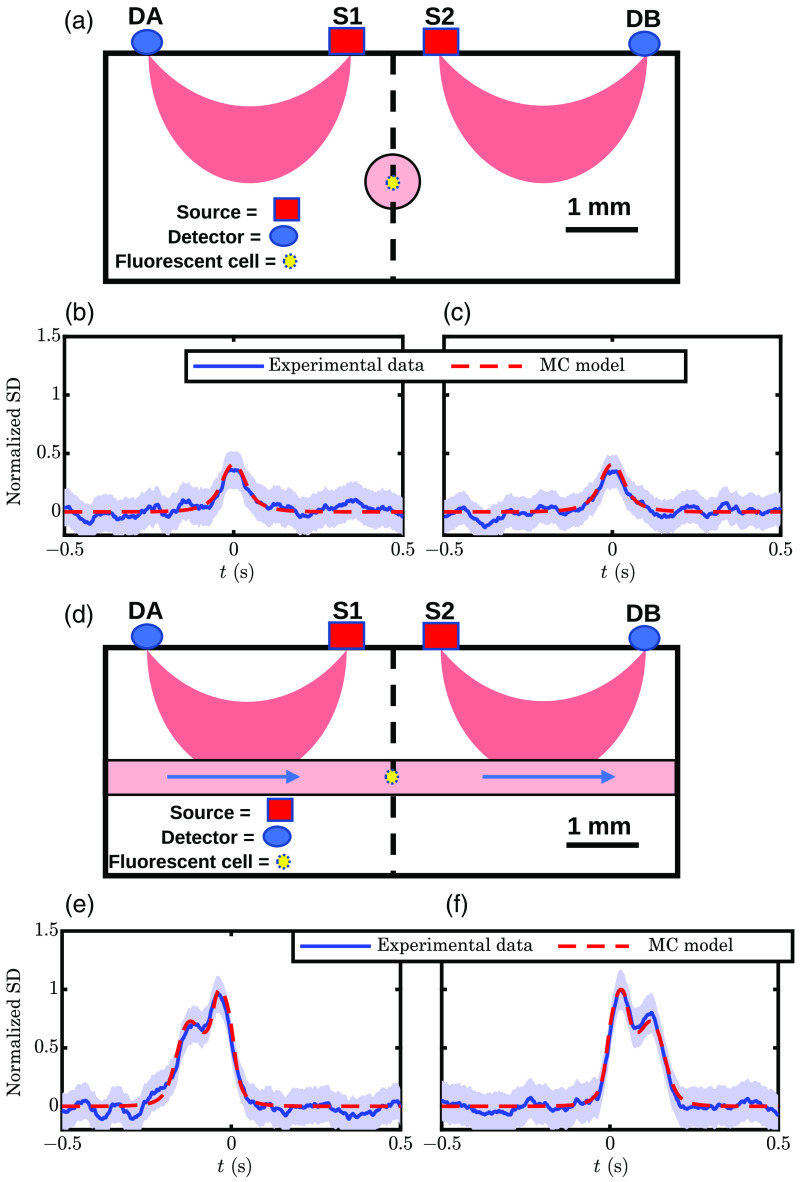
Traces showing a comparison of experimental phantom data and expected results from the MC model. SD traces are normalized so that the mean peak maximum between panels (e) and (f) is one, meaning all subpanels utilize the same normalization factors. (a) Schematic of perpendicular flow case; (b), (c) comparison for perpendicular flow case; (d) schematic of parallel flow case; and (e), (f) comparison for parallel flow case. Note: shaded regions represent the noise level of the experimental data. Parameters: the same as Fig. 2 with a known of 1.5 mm and assumed fluorescent target velocity of $25\;\mathrm{mm}\;s^{-1}$.

We collected experimental DiFC data using a phantom and fluorescent microspheres as described in Sec. 2.3.1. Figure 4 shows sample normalized and smoothed (with the shaded region showing noise) fluorescent microsphere peaks (i.e., detections) flowing 1.50 mm deep in a phantom. DiFC measurements from two SD pairs, A1 and B2, are shown in perpendicular [Figs. 4(a)–4(c)] and parallel [Figs. 4(d)–4(f)] probe to tube configurations. Normalization was performed to set the mean peak maximum of Figs. 4(e) and 4(f) to one while using the same normalization factor for Figs. 4(b) and 4(c). This allows for comparing the relative amplitudes between all traces in Fig. 4.

So that one may relate these normalized values to measured photomultiplier tube (PMT) currents, we provide the experimentally measured amplitudes for this data. For the parallel tube to probe configuration [Figs. 4(e)–4(f)], DiFC peak maximum amplitudes averaged 41 and 38 nA for A1 and B2, respectively. Meanwhile, for the perpendicular tube to probe configuration [Figs. 4(a) and 4(c)], the peak amplitudes averaged 16.64 and 17.22 nA for 1A and 2B, respectively.

We have also overlaid normalized peaks simulated using MC (red dashed lines), using the same normalization method as the experimental data. This type of normalization ensures the mean amplitudes match experimental data for Figs. 4(e) and 4(f) but does not ensure a match for Figs. 4(b) and 4(c) or the individual amplitudes for Figs. 4(e) and 4(f). Additionally, the simulated velocity for the MC data was chosen to match the peak width with the experimental data. For this, a velocity of $25\;\mathrm{mm}\;s^{-1}$ was used. However, since this velocity was assumed for all panels of Fig. 4, a match to the peak width for an individual panel is not ensured. Therefore, a comparison between experimental data and MC simulations should be done against features that were not made to match. These features were the amplitude of the peaks in Figs. 4(b) and 4(c), the amplitude of the lesser second peak in Figs. 4(e) and 4(f), and the overall shape of the signal. With this in mind, we see excellent agreement between the MC simulations and experimental measurements. This is particularly true regarding the doublet peak shown in Figs. 4(e) and 4(f), which is predicted by MC and observed experimentally. Note that the double peak in the parallel configuration is generated by the microsphere passing through high values of W twice, once under the detector and once under the source. Additionally, the relative amplitudes of the doublet are influenced by the relationship between the numerical aperture of the source and detector, reinforcing the choice of a cone instead of a pencil beam for the detector in the MC simulation. In fact, if a pencil beam was chosen for both the source and detector MCs, the match to experimental data would be poor as the doublet peak would have the same maximum for both peaks in the doublet.

## 4 Discussion

### 4.1 Expected Depth

**Fig. 5:**
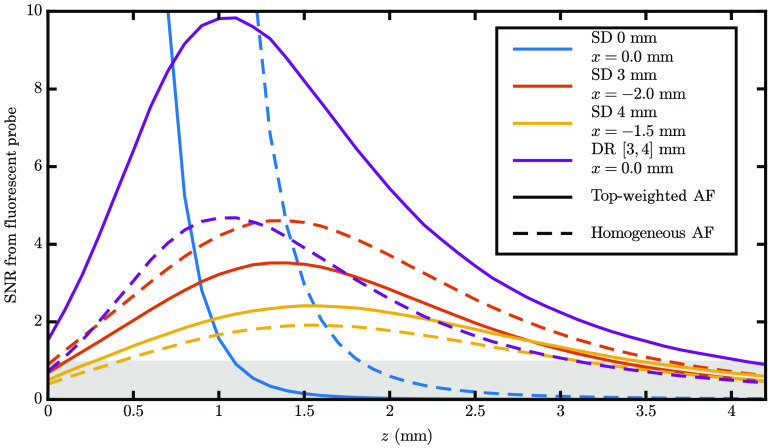
SNR from a fluorescent target below the centroid of the optodes used for each measurement type as a function of depth (z). Colors show different measurement types, and line type shows the AF contributor distribution. Parameters: the same as Fig. 2 with assumed 5% NC noise.

The primary reason for this work is to explore if the DR technique may be applied to DiFC. With this in mind, we must consider what metric to use to compare measurement methods, such as SD versus DR. Since the aim of DiFC is to eventually non-invasively measure blood vessels of humans *in vivo*, the maximum depth that a method can detect a fluorescent target is the metric, which should be used for this comparison. One can determine this depth by examining Fig. 2 and finding the deepest colored region for each data type. However, a line scan in z of Fig. 2, as shown in Fig. 5, makes the relationships more apparent. The x values chosen in Fig. 5 were the centroid of the optodes (e.g., −2.0 x^  mm for 1A); thus the maximum measurable depth can be found by looking at where the curves in Fig. 5 drop below an SNR of one (the gray region). Table 1 summarizes these maximum measurable depths. Note that a suitable vessel to measure in humans, such as the radial artery, is located ∼2 to 4 mm deep.^19^

**Table 1:** Maximum measurable depths of fluorescent target for different measurement types.

	max[z[SNR>1]] (mm)			
	SD ρ=0 mm	SD ρ=3 mm	SD ρ=4 mm	DR $\rho^{\prime }s=[3,4]\;\mathrm{mm}$
Surface-weighted AF contributors	1.0	3.2	3.4	4.0
Homogeneous AF contributors	1.8	3.6	3.1	3.0

Note: SNR, signal-to-noise ratio; SD, single distance; DR, dual ratio; ρ, source–detector distance; and AF, autofluorescence.

Parameters: the same as Fig. 2 with assumed 5% NC noise.

As is evident in Table 1, the depth of DR is heavily affected by the AF contributor distribution. Specifically, if the AF contributor distribution is surface-weighted, DR is the best measurement type, while this is not true for homogeneous. Evidence suggests that the AF contributor distribution is surface-weighted *in vivo*. To investigate the spatial distribution of the AF contributors, we measured the background signal of a mouse as described in Sec. 2.3.2. The mean background with skin was (17400±3500)  nA, whereas the mean background of the bare muscle (without skin) was (9200±540)  nA, which is a reduction of 47%. These results imply that DiFC AF is weighted to superficial tissue layers, and as discussed below, from what we can find in the literature, this seems to be the case qualitatively.

A literature search did not reveal a specific quantification of the spatial distribution of AF contributors. Nevertheless, the available literature on known endogenous fluorophores strongly suggests that these should be weighted more heavily in the skin. Specifically, reduced nicotinamide adenine dinucleotide, flavin adenine dinucleotide, collagen, and elastin fluoresce in the visible (violet and blue) wavelength regions^26^^,^^27^ and, to a lesser degree, in the red and NIR wavelength regions,^28^^,^^29^ the latter being most relevant for the potential use of DR DiFC in humans.^20^ Other significant contributors of tissue AF in the red and NIR wavelength regions are less obvious due to their rarity.^30^ Furthermore, AF in the red and NIR wavelength region may be more complex than visible AF because of the overall reduction in optical scattering and absorption at longer wavelengths.^31^ However, we found lipofuscin,^32^ melanin,^33^^,^^34^ and heme-metabolic compounds, such as porphyrins and bilirubin,^35^^,^^36^ to be reported as AF in the red and NIR wavelength region, all of which are present in the skin. Bilirubin and other heme-metabolic products are mostly known for contributing to liver AF.^37^ However, in the context of DiFC, these compounds may accumulate in the skin from hematomas in the case of injury. Thus the available literature and experimental DiFC measurements in mice (above) suggest that contributors to the background AF in the red and NIR wavelength region for potential DR DiFC are mostly in the skin as opposed to underlying tissue structures.

### 4.2 Consideration of Noise

The noise and signal level used in these simulations was based on experimental data collected at 3 mm SD (Appendix C). However, one noise parameter that cannot be derived from the collected experimental data is the correlation of the noise measured by different optodes. This correlation between optode noises does not affect the SD results but does significantly affect the DR results since it affects how the DR can cancel noise.

We modeled noise as Gaussian random variables (coupling coefficient random variable (C)) that multiplied the theoretical R to yield the measured intensity random variable (I) (Sec. 7.2). A separate independent random variable represents each optode (source or detector) and can be considered noise in the coupling, gain, power, or any other optode-specific noise. This concept, explained in detail in Appendix C, can be summarized by

$$I(R,\sigma_{I}^{2})≃C_{\text{Source}}(1,p_{\text{Source}}{\left(\frac{\sigma_{I}}{R}\right)}^{2})\times C_{\text{detector}}(1,p_{\text{detector}}{\left(\frac{\sigma_{I}}{R}\right)}^{2})\times C_{\mathrm{NC}}(1,p_{\mathrm{NC}}{\left(\frac{\sigma_{I}}{R}\right)}^{2})\times R,$$ (2)

where

$$p_{\text{source}}+p_{\text{detector}}+p_{NC}=1,$$ (3)

using the random variable notation where the first argument is the mean and the second the variance. Since $\sigma_{I}/R\ll 1$, the I defined by the right-hand side of Eq. (2) is an excellent approximation of a random variable with mean R and variance $\sigma_{I}^{2}$ ($I(R,\sigma_{I}^{2})$). The DR cancels all C’s associated with specific optodes [Eq. (33)] but not the non-cancelable (NC) C. NC is named such since it is the noise not canceled by DR. From this, we see that the parameter controlling the amount of noise that propagates into DR is $p_{\mathrm{NC}}$, which is the fraction of the variance that is NC by DR.

In all the above results in this work, $p_{\mathrm{NC}}$ was assumed to be 5%. Note that we do not know the physical origin of this noise if all SDs are acquired simultaneously, so we apply this noise to noise introduced from multiplexing. We expect that advanced multiplexing schemes may alleviate this noise (Sec. 4.3) but do not know what it is for the current system, so we assumed what we consider a reasonable value of 5%.

**Fig. 6:**
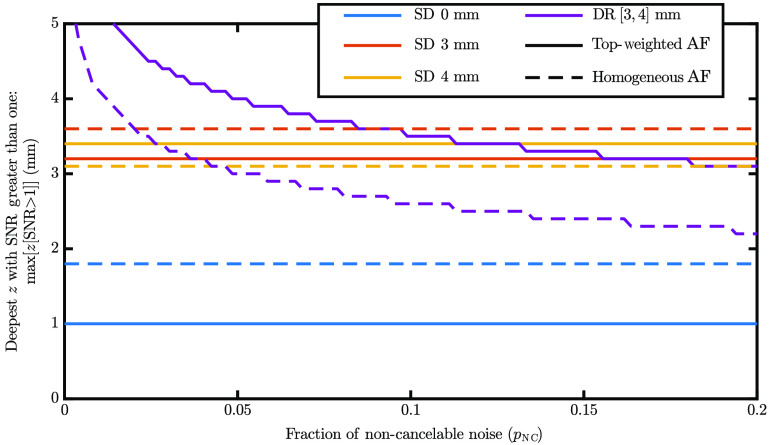
The deepest fluorescent target that each data type can measure (SNR > 1) as a function of the fraction of NC (by DR) noise ($p_{\mathrm{NC}}$). Colors show the data type, and line type shows the distribution of AF contributors. Note: for a definition of see Sec. 7.2 or Eq. (2). Parameters: the same as Fig. 2.

To experiment with the effect of $p_{\mathrm{NC}}$, we varied its value and determined the maximum measurable depth (where SNR is >1) for each data type in Fig. 6. Note that all SD measurements experience the same noise regardless of $p_{\mathrm{NC}}$’s value, and only DR is affected. This is because the model was designed so that the total noise is the same regardless of the $p_{\mathrm{NC}}$ value. From Fig. 6, we can find the allowable $p_{\mathrm{NC}}$, which makes DR sense deeper than the deepest SD modeled. For the simulation with surface-weighted AF contributions, this is 12%, while for the homogeneous case, it is 2%. This further emphasizes the advantage DR has in the surface-weighted AF medium. Additionally, this guides the amount of $p_{\mathrm{NC}}$, which a system can have for DR to have an advantage over SD.

### 4.3 Future Implementation

The experimental and computational data presented here were based on measurements using SD or single-ratio (SR) (not presented for brevity) measurements taken in series using our existing DiFC instrument.^1^^,^^3^ In practice, implementation of a DR for DiFC would require the construction of a new DiFC instrument capable of making simultaneous measurements with two sources and two detectors as in Fig. 1(c). Several optical designs would enable this. For example, we could frequency encode the two laser sources by modulating them at different frequencies and demodulating the fluorescence signals measured by the two detectors to separate the contributions from the two sources (frequency multiplexing). Likewise, we could alternately illuminate the two laser sources (S1 and S2) in an on-off pattern (time multiplexing). Assuming a peak width of 10 ms *in vivo*,^1^ this should be achievable by time multiplexing the laser output at about 1 kHz.

Fast time multiplexing or frequency multiplexing would be desirable because the DR strategy would inherently cancel out signal drift and noise. DR cancelable artifacts are associated with a multiplicative factor applied to a source or detector that does not change within the multiplexing cycle (Sec. 7.2.1). This could include instrument drift or coupling of the sources and detectors to the skin surface. However, this approach cannot cancel some sources of random noise in the signal (Sec. 7.2.1 and Fig. 6). In principle, using modulated laser sources and lock-in detection for frequency multiplexing would improve the SNR of detected peaks in the presence of NC noise. Furthermore, as shown in Figs. 3 and 4, the DR DiFC measurement using a parallel configuration [Fig. 1(b)] yielded a unique double-peak shape. Therefore, we expect that using matched filters (or machine learning methods based on signal temporal shape) could improve peak detection. DR noise would also be suppressed when the two ρs are as different as possible.^22^ Therefore, custom-designed optical fiber bundles may be convenient for delivering and collecting light in a DR DiFC instrument. For instance, we can design a new DiFC optical fiber bundle that incorporates multiple source fibers with symmetric separations and large detector areas.

Finally, we note that this work is the first case of dual slope/DR applied to fluorescence in general, not only to DiFC. Dual slope/DR was first developed for NIR spectroscopy (NIRS) and measurement of changes in $\mu_{a}(\Delta \mu_{a})$.^5^^,^^7^ Now, with the methods presented in this work, we believe dual slope/DR may also be applied to diffuse fluorescence spectroscopy and tomography.^27^^,^^35^^,^^38^^–^^41^ Dual slope/DR should lend itself well to any application, which aims to measure changes in fluorescence (i.e., instead of $\Delta \mu_{a}$ as it was used for in NIRS). Thus, we open the door to future work regarding dual slope/DR fluorescence.

## 5 Conclusion

This work explored the feasibility of using DR for DiFC. We accomplished this by running MC models of the expected DR SNR based on noise and fluorescence parameters extracted from experimental DiFC data. From this exploration, we modify two key factors that control whether DR is beneficial to DiFC or not. The first is the distribution of AF contributors *in vivo*, with surface-weighted giving DR methods an advantage. Experiments on mice and a literature search suggest that the *in vivo* AF contributor distribution is concentrated in the skin, suggesting that DR will have an advantage over traditional SD methods. The second key parameter is the portion of NC (by DR) noise in the measurement. This noise is not associated with multiplicative optode factors (e.g., tissue coupling, source power, or detector efficiency), which are constant within a multiplexing cycle. A DiFC instrument designed for DR would need to be built to explore and address this. A future DiFC instrument may achieve the DR measurement by time- or frequency multiplexing leading to the required level of NC noise. Therefore, this work laid the groundwork to identify the key parameters of concern and aid in designing a DR DiFC instrument.

## 6 Appendix A: Definition of Measurement Types

### 6.1 Single Distance

The raw measurement from a DiFC system is the I measured between a source and a detector that we also refer to as the SD here. Using this raw measurement, we define the change in the SD (ΔSD) as the background-subtracted I as follows:

$$\Delta \mathrm{SD}(t)=I(t)-I_{0},$$ (4)

where ΔSD is expressed as a function of time (t) and $I_{0}$ is the average or I over a baseline t. $I_{0}$ could be found in various ways, for example: based on an initial measurement or an average over a preceding time period, regardless ΔSD represents a change in I over t. Here the primary measurement parameter is the source–detector distance (ρ), in this work, SDs are evaluated at ρ of either 3 or 4 mm.

### 6.2 Single Ratio

Next, we define the SR, which is a ratio of Is measured at long and short ρs:

$$\mathrm{SR}=\frac{I_{l}}{I_{s}},$$ (5)

where the l or s subscripts represent measurements at either long or short ρs, respectively. Using Eq. (5), we next express the change in the SR (ΔSR):

$$\Delta \mathrm{SR}(t)=\frac{I_{l}(t)}{I_{s}(t)}-\frac{I_{l,0}}{I_{s,0}},$$ (6)

which, as with ΔSD, is also expressed as a function of time. The primary parameters for SR are the long and short ρs, which are 3 and 4 mm in this work.

### 6.3 Dual Ratio

Finally, we define the DR, which is the geometric mean of two SRs related through geometric requirements. These requirements can be summarized by stating that optodes that contribute to the short ρ for one SR contribute to the long ρ for the other SR in a DR and vice versa. Suppose we have two SRs named ${\mathrm{SR}}_{I}$ and ${\mathrm{SR}}_{\mathrm{II}}$. Naming sources as numbers and detectors as letters, we can say that ${\mathrm{SR}}_{I}$ contains one detector (A) and two sources (1 and 2) such that the short ρ is obtained between 1 and A and the long ρ between 2 and A [Fig. 1(d)]. Now consider ${\mathrm{SR}}_{\mathrm{II}}$ to be made of detector B and the same two sources 1 and 2 with the short ρ being between B and 2 and the long ρ between B and 1. Now notice that for ${\mathrm{SR}}_{I}$ 1 forms the short ρ but for ${\mathrm{SR}}_{\mathrm{II}}$ 1 forms the long ρ. Similarly, for ${\mathrm{SR}}_{\mathrm{II}}$ 2 forms the short ρ but for ${\mathrm{SR}}_{I}$ 2 forms the long ρ. Therefore, the geometric requirements are satisfied, and the geometric mean of ${\mathrm{SR}}_{I}$ and ${\mathrm{SR}}_{\mathrm{II}}$ form a DR:

$$\mathrm{DR}=\sqrt{{\mathrm{SR}}_{I}{\mathrm{SR}}_{\mathrm{II}}}=\sqrt{\frac{I_{l,I}I_{l,\mathrm{II}}}{I_{s,I}I_{s,\mathrm{II}}}},$$ (7)

and thus change in the DR (ΔDR) can we written as

$$\Delta \mathrm{DR}(t)=\sqrt{\frac{I_{l,I}(t)I_{l,\mathrm{II}}(t)}{I_{s,I}(t)I_{s,\mathrm{II}}(t)}}-\sqrt{\frac{I_{l,I,0}I_{l,\mathrm{II},0}}{I_{s,I,0}I_{s,\mathrm{II},0}}}.$$ (8)

Similar to SR, the parameters that are important for DR are the short and long ρs. However, in this case, there are two short and two long ρs. In this work, both short are considered to be 3 mm and both long 4 mm.

## 7 Appendix B: Theory

### 7.1 Modeling Fluorescent Reflectance

To model the detected fluorescent reflectance (R), we must consider two processes: first, the delivery of power to the fluorophores and second, the transport of emitted light from the fluorophores to the detector.

#### 7.1.1 Dependence on fluorophore position

The expression for the power volume density (Q) absorbed by a fluorophore [$Q_{\mathrm{fl},\mathrm{ab}}({\vec{r}}_{\mathrm{fl}})$, unit of $\mathrm{mW}\;{\mathrm{mm}}^{-3}$] at the position vector $\left(\vec{r}\right)$ of the fluorophore $\left({\vec{r}}_{\mathrm{fl}}\right)$ can be written as

$$Q_{\mathrm{fl},\mathrm{ab}}({\overset{\to }{r}}_{\mathrm{fl}})=\Phi ({\overset{\to }{r}}_{\mathrm{src}}\to {\overset{\to }{r}}_{\mathrm{fl}})\mu_{a,\mathrm{fl}}({\overset{\to }{r}}_{\mathrm{fl}}),$$ (9)

where $\mu_{a,\mathrm{fl}}$ is the absorption coefficient $\left(\mu_{a}\right)$ for the fluorophore (unit of ${\mathrm{mm}}^{-1}$) and $\Phi ({\vec{r}}_{\mathrm{src}}\to {\vec{r}}_{\mathrm{fl}})$ is the fluence rate (Φ) (using the excitation optical wavelength (λ) optical properties) from a pencil beam at the source position $\left({\vec{r}}_{\mathrm{src}}\right)$ to the fluorophore (unit of $\mathrm{mW}\;{\mathrm{mm}}^{-2}$). Next, we consider the Q emitted by the fluorophore [$Q_{\mathrm{fl},\mathrm{em}}({\overset{\to }{r}}_{\mathrm{fl}})$, unit of $\mathrm{mW}\;{\mathrm{mm}}^{-3}$]:

$$Q_{\mathrm{fl},\mathrm{em}}({\vec{r}}_{\mathrm{fl}})=Q_{\mathrm{fl},\mathrm{ab}}({\vec{r}}_{\mathrm{fl}})\eta ({\vec{r}}_{\mathrm{fl}}),$$ (10)

where fluorescence efficiency (η) represents the proportion of absorbed power converted to fluorescence. Thinking of the medium as voxelized with voxels with volume (V), we volumetrically integrate Eq. (10) over V (unit of ${\mathrm{mm}}^{3}$) to yield the power (P) emitted by the fluorophore at position ${\vec{r}}_{\mathrm{fl}}$ inside the voxel [$P_{\mathrm{fl},\mathrm{em}}({\overset{\to }{r}}_{\mathrm{fl}})$, unit of mW]:

$$P_{\mathrm{fl},\mathrm{em}}({\overset{\to }{r}}_{\mathrm{fl}})=Q_{\mathrm{fl},\mathrm{ab}}({\overset{\to }{r}}_{\mathrm{fl}})\eta ({\overset{\to }{r}}_{\mathrm{fl}})V.$$ (11)

Finally, we find the fluorescent R from the fluorophore within V at ${\vec{r}}_{\mathrm{fl}}$, which is excited by a pencil beam at ${\overset{\to }{r}}_{\mathrm{src}}$ and detected at ${\vec{r}}_{\det}$ ($R_{\mathrm{fl}}({\overset{\to }{r}}_{\mathrm{src}}\to {\overset{\to }{r}}_{\mathrm{fl}}\to {\overset{\to }{r}}_{\det})$ (unit of $\mathrm{mW}\;{\mathrm{mm}}^{-2}$). $R_{\mathrm{fl}}({\vec{r}}_{\mathrm{src}}\to {\vec{r}}_{\mathrm{fl}}\to {\vec{r}}_{\det})$ is obtained by multiplying $P_{\mathrm{fl},\mathrm{em}}({\overset{\to }{r}}_{\mathrm{fl}})$ by the R Green’s function (using the emission λ optical properties) from ${\vec{r}}_{\mathrm{fl}}$ to ${\vec{r}}_{\det}$ [$R_{\text{Green}}({\overset{\to }{r}}_{\mathrm{fl}}\to {\overset{\to }{r}}_{\det})$, unit of ${\mathrm{mm}}^{-2}$]:

$$R_{\mathrm{fl}}({\overset{\to }{r}}_{\mathrm{src}}\to {\overset{\to }{r}}_{\mathrm{fl}}\to {\overset{\to }{r}}_{\det})=P_{\mathrm{fl},\mathrm{em}}({\overset{\to }{r}}_{\mathrm{fl}})R_{\text{Green}}({\overset{\to }{r}}_{\mathrm{fl}}\to {\overset{\to }{r}}_{\det}).$$ (12)

To simplify these expressions, we rewrite them to yield:

$$R_{\mathrm{fl}}({\overset{\to }{r}}_{\mathrm{src}}\to {\overset{\to }{r}}_{\mathrm{fl}}\to {\overset{\to }{r}}_{\det})=\Phi ({\overset{\to }{r}}_{\mathrm{src}}\to {\overset{\to }{r}}_{\mathrm{fl}})R_{\text{Green}}({\overset{\to }{r}}_{\mathrm{fl}}\to {\overset{\to }{r}}_{\det})V\mu_{a,fl}({\overset{\to }{r}}_{\mathrm{fl}})\eta ({\overset{\to }{r}}_{\mathrm{fl}}).$$ (13)

Note that $R_{\text{Green}}({\overset{\to }{r}}_{\mathrm{fl}}\to {\overset{\to }{r}}_{\det})$ is rather difficult to calculate using methods, such as MC, so we may use the Green’s function of the Φ (using the emission λ optical properties) with a source placed at ${\vec{r}}_{\det}$ [$\Phi_{\text{Green}}({\overset{\to }{r}}_{\det}\to {\overset{\to }{r}}_{\mathrm{fl}})$, unit of ${\mathrm{mm}}^{-2}$] as an approximation according to the adjoint method.^24^ Additionally, we note that $\Phi ({\overset{\to }{r}}_{\mathrm{src}}\to {\overset{\to }{r}}_{\mathrm{fl}})$ can be expressed as the Green’s function for Φ [$\Phi_{\text{Green}}({\overset{\to }{r}}_{\mathrm{src}}\to {\overset{\to }{r}}_{\mathrm{fl}})$, unit of ${\mathrm{mm}}^{-2}$] multiplied by the P of the source ($P_{\mathrm{src}}$, unit of mW). These approximations and substitutions yield:

$$R_{\mathrm{fl}}({\overset{\to }{r}}_{\mathrm{src}}\to {\overset{\to }{r}}_{\mathrm{fl}}\to {\overset{\to }{r}}_{\det})\approx P_{\mathrm{src}}\Phi_{\text{Green}}({\overset{\to }{r}}_{\mathrm{src}}\to {\overset{\to }{r}}_{\mathrm{fl}})\Phi_{\text{Green}}({\overset{\to }{r}}_{\det}\to {\overset{\to }{r}}_{\mathrm{fl}})V\mu_{a,\mathrm{mfl}}({\overset{\to }{r}}_{\mathrm{fl}})\eta ({\overset{\to }{r}}_{\mathrm{fl}}).$$ (14)

We remind that $\Phi_{\text{Green}}({\overset{\to }{r}}_{\mathrm{src}}\to {\overset{\to }{r}}_{\mathrm{fl}})$ and $\Phi_{\text{Green}}({\overset{\to }{r}}_{\det}\to {\overset{\to }{r}}_{\mathrm{fl}})$ are the Green’s functions calculated at the excitation and emission wavelengths, respectively. In this work, however, we assume the optical properties at the two wavelengths to be the same. Last, we define the fluorescence Jacobian (W),^20^ which is a helpful parameter in understanding how the $R_{\mathrm{fl}}({\vec{r}}_{\mathrm{src}}\to {\vec{r}}_{\mathrm{fl}}\to {\vec{r}}_{\det})$ depends on the spatial location of the fluorophore:

$$W({\overset{\to }{r}}_{\mathrm{src}}\to {\overset{\to }{r}}_{\mathrm{fl}}\to {\overset{\to }{r}}_{\det})=\Phi_{\text{Green}}({\overset{\to }{r}}_{\mathrm{src}}\to {\overset{\to }{r}}_{\mathrm{fl}})\Phi_{\text{Green}}({\overset{\to }{r}}_{\det}\to {\overset{\to }{r}}_{\mathrm{fl}})V,$$ (15)

$$R_{\mathrm{fl}}({\vec{r}}_{\mathrm{src}}\to {\vec{r}}_{\mathrm{fl}}\to {\vec{r}}_{\det})\approx P_{\mathrm{src}}W({\vec{r}}_{\mathrm{src}}\to {\vec{r}}_{\mathrm{fl}}\to {\vec{r}}_{\det})\mu_{a,\mathrm{fl}}({\vec{r}}_{\mathrm{fl}})\eta ({\vec{r}}_{\mathrm{fl}}).$$ (16)

In fact if $\mu_{a,\mathrm{fl}}({\vec{r}}_{\mathrm{fl}})$ and $\eta ({\vec{r}}_{fl})$ are both spatially independent, $W({\vec{r}}_{\mathrm{src}}\to {\vec{r}}_{\mathrm{fl}}\to {\vec{r}}_{\det})$ (unit of ${\mathrm{mm}}^{-1}$) is directly proportional to $R_{fl}({\vec{r}}_{\mathrm{src}}\to {\vec{r}}_{\mathrm{fl}}\to {\vec{r}}_{\det})$ and representative of how the spatial location of the fluorophore contributes to the detected fluorescence.

#### 7.1.2 Fluorescent reflectance from multiple fluorophores

It is assumed that the R measured from each voxel containing a fluorophore may be linearly combined. Therefore:

$$R_{\mathrm{fl}}({\vec{r}}_{\mathrm{src}}\to \Sigma {\vec{r}}_{\mathrm{fl}}\to {\vec{r}}_{\det})\approx P_{\mathrm{src}}\Sigma_{i}W({\vec{r}}_{\mathrm{src}}\to {\vec{r}}_{\mathrm{fl},i}\to {\vec{r}}_{\det})\mu_{a,\mathrm{fl}}({\vec{r}}_{\mathrm{fl},i})\eta ({\vec{r}}_{\mathrm{fl},i}),$$ (17)

and if $\mu_{a,\mathrm{fl}}({\vec{r}}_{\mathrm{fl},i})$ and $\eta ({\vec{r}}_{\mathrm{fl},i})$ (where the subscript i indicates the i’th voxel) are spatially constant:

$$R_{\mathrm{fl}}({\overset{\to }{r}}_{\mathrm{src}}\to \Sigma {\overset{\to }{r}}_{\mathrm{fl}}\to {\overset{\to }{r}}_{\det})\approx P_{\mathrm{src}}\mu_{a,\mathrm{fl}}\eta W({\overset{\to }{r}}_{\mathrm{src}}\to \Sigma {\overset{\to }{r}}_{\mathrm{fl}}\to {\overset{\to }{r}}_{\det}),$$ (18)

where

$$W({\overset{\to }{r}}_{\mathrm{src}}\to \Sigma {\overset{\to }{r}}_{\mathrm{fl}}\to {\overset{\to }{r}}_{\det})=V\Sigma_{i}\Phi_{\text{Green}}({\overset{\to }{r}}_{\mathrm{src}}\to {\overset{\to }{r}}_{\mathrm{fl},i})\Phi_{\text{Green}}({\overset{\to }{r}}_{\det}\to {\overset{\to }{r}}_{\mathrm{fl},i}).$$ (19)

The background R is a case where the sum of all the AF fluorophores from every voxel must be considered. In this case, Eq. (17) is considered for a heterogeneous distribution of AF $\mu_{a}$ or η (i.e., AF contributions) and Eq. (18) for a homogeneous AF $\mu_{a}$ or η. Any signal from a target fluorescent target is then added to this background R using Eq. (16). This yields an expression for the fluorescent R considering contributions from both the target fluorescent target at ${\overset{\to }{r}}_{\text{target}}$ and all AF contributors:

$$R({\overset{\to }{r}}_{\text{target}})\approx P_{\mathrm{src}}(\Sigma_{i}W({\overset{\to }{r}}_{i})\mu_{a,\mathrm{AF}}({\overset{\to }{r}}_{i})\eta_{\mathrm{AF}}({\overset{\to }{r}}_{i})+W({\overset{\to }{r}}_{\text{target}})\mu_{a,\text{target}}({\overset{\to }{r}}_{\text{target}})\eta_{\text{target}}({\overset{\to }{r}}_{\text{target}})).$$ (20)

Here we have dropped the notation showing the source and detector $\vec{r}$ for brevity. Notice that the background R expressed as $R_{0}$ is the AF term, such that:

$$R_{0}\approx P_{\mathrm{src}}\Sigma_{i}W({\overset{\to }{r}}_{i})\mu_{a,\mathrm{AF}}({\overset{\to }{r}}_{i})\eta_{\mathrm{AF}}({\overset{\to }{r}}_{i}),$$ (21)

and

$$R({\overset{\to }{r}}_{\text{target}})\approx P_{\mathrm{src}}W({\overset{\to }{r}}_{\text{target}})\mu_{a,\text{target}}({\overset{\to }{r}}_{\text{target}})\eta_{\text{target}}({\overset{\to }{r}}_{\text{target}})+R_{0}.$$ (22)

For simulations these expressions, namely Eqs. (20) and (21), are used as the simulated Rs to obtained simulated measured I using methods in Sec. 7.2. These simulated measured Is can then be used with the expressions in Appendix A to obtain simulated measurement types SD and DR.

### 7.2 Simulating Noise and Coupling Coefficients

#### 7.2.1 Coupling coefficients

We assume that the measured I is related to the theoretical R through multiplicative coupling coefficients (C’s) typically associated with each optode. Notice that we distinguish between the measured and theoretical values by naming the former I and the latter R. As such, the equations in Appendix A show measurement types derived from measured (or simulated measured) Is, whereas the expressions in Appendix B show calculation of theoretical Rs. The use of Cs in this section serves to connect I and R. Therefore, C may take the proper units to convert the measured I (measured in nA from a PMT for example) to the unit of R ($\mathrm{mW}\;{\mathrm{mm}}^{-2}$). Additionally, if noise is considered, C may be a random variable representing the noise that confounds I.

Given the optode arrangement in Fig. 1(c), we have two sources (1 and 2) and two detectors (A and B). Therefore, four measurements of I between a source and detector are possible: $I_{1A}$, $I_{1B}$, $I_{2A}$, and $I_{2B}$, which are related to R through Cs as follows:

$$I_{1A}=C_{1}C_{A}C_{\mathrm{NC},\alpha }R_{1A},$$ (23)

$$I_{1B}=C_{1}C_{B}C_{\mathrm{NC},\beta }R_{1B},$$ (24)

$$I_{2A}=C_{2}C_{A}C_{\mathrm{NC},\gamma }R_{2A},$$ (25)

$$I_{2B}=C_{2}C_{B}C_{\mathrm{NC},\delta }R_{2B},$$ (26)

where the Cs with 1, 2, A, or B subscripts are associated with the corresponding optode (source or detector). The $C_{\mathrm{NC}}$s are NC coupling factors or noise not associated with a specific optode and from an unknown source. NC refers to these $C_{\mathrm{NC}}$s not being canceled by the DR measurement, as is shown later in this section. Notice that there is also a unique $C_{\mathrm{NC}}$ (shown with Greek subscripts) for each I measurement.

#### 7.2.2 Noise

Now we consider noise and thus model the Cs as Gaussian coupling coefficient random variables (C’s). In the following, we assume I and R have the same units and scale (making C unit-less and mean one); however, in reality, a coefficient converting R to I multiplies the C’s. We assume that the intensity noise $\left(\sigma_{I}\right)$ is proportional to the R such that relative noise $\left(\sigma_{\mathrm{rel}}\right)$ is constant across different I’s, where

$$\sigma_{\mathrm{rel}}=\sigma_{I}/R.$$ (27)

Therefore, the intensity random variable (I) from Eq. (2) has the mean value R and variance $\sigma_{I}^{2}$ ($I(R,\sigma_{I}^{2})=I(R,\sigma_{\mathrm{rel}}^{2}R^{2})$). This can be modeled by taking the mean of all C’s one and the sum of the three C’s variance who contribute to a single I equal to $\sigma_{I}^{2}/R^{2}$. To model this, we introduce two parameters, the fraction of noise contributed from each optode ($p_{\mathrm{opt}}$) and the fraction of NC noise ($p_{\mathrm{NC}}$). Thus we can model the I noise as

$$I_{1A}(R_{1A},\sigma_{I1A}^{2})=C_{1}(1,p_{\mathrm{opt}}\sigma_{\mathrm{rel}}^{2})C_{A}(1,p_{\mathrm{opt}}\sigma_{\mathrm{rel}}^{2})C_{\mathrm{NC},\alpha }(1,p_{\mathrm{NC}}\sigma_{\mathrm{rel}}^{2})R_{1A},$$ (28)

$$I_{1B}(R_{1B},\sigma_{I1B}^{2})=C_{1}(1,p_{\mathrm{opt}}\sigma_{\mathrm{rel}}^{2})C_{B}(1,p_{\mathrm{opt}}\sigma_{\mathrm{rel}}^{2})C_{\mathrm{NC},\beta }(1,p_{\mathrm{NC}}\sigma_{\mathrm{rel}}^{2})R_{1B},$$ (29)

$$I_{2A}(R_{2A},\sigma_{I2A}^{2})=C_{2}(1,p_{\mathrm{opt}}\sigma_{\mathrm{rel}}^{2})C_{A}(1,p_{\mathrm{opt}}\sigma_{\mathrm{rel}}^{2})C_{\mathrm{NC},\gamma }(1,p_{\mathrm{NC}}\sigma_{\mathrm{rel}}^{2})R_{2A},$$ (30)

$$I_{2B}(R_{2B},\sigma_{I2B}^{2})=C_{2}(1,p_{\mathrm{opt}}\sigma_{\mathrm{rel}}^{2})C_{B}(1,p_{\mathrm{opt}}\sigma_{\mathrm{rel}}^{2})C_{\mathrm{NC},\delta }(1,p_{\mathrm{NC}}\sigma_{\mathrm{rel}}^{2})R_{2B},$$ (31)

where

$$2p_{\mathrm{opt}}+p_{\mathrm{NC}}=1.$$ (32)

In the simulations shown in this work, the C’s takes the form of a Gaussian distribution.

#### 7.2.3 Propagation of coupling coefficients and noise to dual ratio

This work presents the results of SD and DR. Therefore, it is helpful to understand how these noise models affect each. For the case of SD, coupling and noise affect the measurement in the same way as I is shown to be involved above. However, for DR, all coupling and noise are canceled except for the NC component. To show this, we rewrite Eq. (7) considering the optodes in Fig. 1(d) but replacing Is with Cs and Rs:

$$\mathrm{DR}=\sqrt{\frac{C_{1}C_{B}C_{\mathrm{NC},\beta }R_{1B}C_{2}C_{A}C_{\mathrm{NC},\gamma }R_{2A}}{C_{1}C_{A}C_{\mathrm{NC},\alpha }R_{1A}C_{2}C_{B}C_{\mathrm{NC},\delta }R_{2B}}}=\sqrt{\frac{C_{\mathrm{NC},\beta }C_{\mathrm{NC},\gamma }}{C_{\mathrm{NC},\alpha }C_{\mathrm{NC},\delta }}}\sqrt{\frac{R_{1B}R_{2A}}{R_{1A}R_{2B}}},$$ (33)

leading to

$$\mathrm{DR}=\sqrt{\frac{C_{\mathrm{NC},\beta }C_{\mathrm{NC},\gamma }}{C_{\mathrm{NC},\alpha }C_{\mathrm{NC},\delta }}}{\mathrm{DR}}_{\mathrm{theo}},$$ (34)

where ${\mathrm{DR}}_{\mathrm{theo}}$ is the theoretical DR. Therefore, the measured and theoretical DR are equal if $p_{\mathrm{NC}}=0$. Additionally, this demonstrates that DR eliminates Cs associated with multiplicative factors belonging to the optodes, and only the NC $C_{\mathrm{NC}}$s remain in the DR measurement.

## 8 Appendix C: Simulation Parameters from Experimental Data

### 8.1 Determining Background and Peak Fluorescence Coefficients

The experimental data from Sec. 2.3.1 was used to calculate η and $\mu_{a}$ for both background and target ($\eta_{\mathrm{AF}}\mu_{a,\mathrm{AF}}$ and $\eta_{\text{target}}\mu_{a,\text{target}}$, respectively). This allowed for the simulated measurement which we present. The PMTs output measurement was I in nA; therefore, it was assumed that the Cs in Eq. (23) together had the units of $W\;{\mathrm{mm}}^{-2}\;{\mathrm{nA}}^{-1}$ making R have the unit of $W\;{\mathrm{mm}}^{-2}$. We assumed this value to be $271\times 10^{-15}\;W\;{\mathrm{mm}}^{-2}\;{\mathrm{nA}}^{-1}$ based on detector gain ($1\times 10^{4}$), detector area ($0.565\;{\mathrm{mm}}^{2}$), and λ (810 nm). However, since this value cancels whenever a unit-less quantity is calculated (such as SNR), we stress that what we assume for this value does not matter for the results; we simply assume one to match units between the experimental data and MC. As for MC parameters for calculation of W, we assume a $\mu_{a}$ of $0.002\;{\mathrm{mm}}^{-1}$, a scattering coefficient $\left(\mu_{s}\right)$ of $7\;{\mathrm{mm}}^{-1}$, and a index of refraction (n) of 1.37. The experimental data collected for these calculations were measured at a ρ of 3 mm and a source P of 75 mW. Finally, the MC used a voxel size of 0.1  mm×0.1  mm×0.1  mm and launched $1\times 10^{9}$ photons.

#### 8.1.1 Background autofluorescence

To find the background I/R, the moving mean using a 1 s window (with a sample rate of 2 kHz) was found for all experimental data, with outliers removed (defining outliers as values more than three scaled mean absolute deviations from the median). Then the median of all moving mean values was found and taken as the background. Using this method, we found an average measured background at ρ=3  mm, of 170 nA or $46.1\;\mathrm{pW}\;{\mathrm{mm}}^{-2}$.

When assuming the AF contributor distribution to be heterogeneous and surface-weighted, we model the $\eta_{\mathrm{AF}}$ as exponentially decaying as one goes deeper into the medium:

ηAF,het(r→)=eln(1/2)(r→·z^)/0.1  mm. (35)

Then rewrite Eq. (21) as follows:

$$R_{0}\approx P_{\mathrm{src}}\mu_{a,\mathrm{AF},\mathrm{het}}\Sigma_{\mathrm{All}\vec{r}}W(\vec{r})e^{\ln(1/2)(\vec{r}\cdot \hat{z})/0.1\;\mathrm{mm}}$$ (36)

and solve for $\mu_{a,\mathrm{AF}}$:

μa,AF,het≈R0PsrcΣAllr→W(r→)eln(1/2)(r→·z^)/0.1  mm. (37)

With this method, we found that μa,AF,hetηAF,het=228×10−9  mm−1×eln(1/2)(r→·z^)/0.1  mm for the measured background AF.

In the case of homogeneous AF contributors, we instead rewrite and solve Eq. (21) as

$$\mu_{a,\mathrm{AF},\mathrm{hom}}\eta_{\mathrm{AF},\mathrm{hom}}\approx \frac{R_{0}}{P_{\mathrm{src}}\Sigma_{\text{All}\overset{\to }{r}}W(\overset{\to }{r})},$$ (38)

which yields $\mu_{a,\mathrm{AF},\mathrm{hom}}\eta_{\mathrm{AF},\mathrm{hom}}=6.81\times 10^{-9}\;{\mathrm{mm}}^{-1}$, assuming the optical properties used above for the heterogeneous case.

These values of $\mu_{a,\mathrm{AF}}\eta_{\mathrm{AF}}$, whether homogeneous or heterogeneous, were used for the simulation results presented. Specifically, by implementing their values in Eqs. (21)–(22) to yield the simulated R.

#### 8.1.2 Fluorescence peak amplitude

For the peak amplitude, we found peaks with an amplitude at least five times the noise (calculated in Sec. 8.2) and at least 1 s apart. Considering the peak amplitude as the background-subtracted measurement, we found a mean amplitude of 47.3 nA or $12.8\;\mathrm{pW}\;{\mathrm{mm}}^{-2}$. This was for ρ=3  mm and a target depth of 1.5 mm.

Then rewriting Eq. (22) as

$$\mu_{a,\text{target}}\eta_{\text{target}}\approx \frac{R_{\text{peak}}-R_{0}}{P_{\mathrm{src}}\;\max_{\overset{\to }{r}}(W(\overset{\to }{r}))},$$ (39)

we can find the $\mu_{a,\text{target}}\eta_{\text{target}}$ by assuming that the peak maximum occurred when the target was at the location with the highest sensitivity (W). Using this method, we found $\mu_{a,\text{target}}\eta_{\text{target}}=43.3\times 10^{-6}\;{\mathrm{mm}}^{-1}$. This value, with the ones for the AF above, was used for the simulations in this paper using Eq. (22).

### 8.2 Measurement of Noise

To find the noise of the I/R, the moving standard deviation using a 1 s window (with a sample rate of 2 kHz) was found for all experimental data, with outliers removed (defining outliers as values more than three scaled mean absolute deviations from the median), similar to the method used to find the background. Then the median of all moving standard deviation values was found and taken as the noise. Using this method, we found an average measured noise at ρ=3  mm of 5.29 nA or $1.43\;\mathrm{pW}\;{\mathrm{mm}}^{-2}$.

Considering the peak amplitude found to be 47.3 nA or $12.8\;\mathrm{pW}\;{\mathrm{mm}}^{-2}$, the SNR at SD ρ=3  mm was found to be 8.95 on average. The $\sigma_{\mathrm{rel}}$ [Eq. (27)] was the key parameter used to simulate noise for other distances. Given the background value of 170 nA or $46.1\;\mathrm{pW}\;{\mathrm{mm}}^{-2}$, this results in a $\sigma_{\mathrm{rel}}$ of 0.031. This value was used for the noise simulations as described in Sec. 7.2 leading to the SNR results presented.

## Data, Materials, and Code Availability

Supporting code and data can be found at: github.com/DOIT-Lab/DOIT-Public/tree/master/DualRatio_FluorescenceFlowCytometry. Any additional supporting code and data are available from the authors upon reasonable request.
